# Introduction to *topiCS* Volume 14, Issue 4

**DOI:** 10.1111/tops.12627

**Published:** 2022-10-04

**Authors:** Andrea Bender

**Affiliations:** ^1^ Executive Editor

**Keywords:** Navigation, Individual differences, Spatial cognition

1

In the recent debate about the unity and integration of cognitive science (Núñez et al., 2019, 2020; see also commentaries in *topiCS 11:4*, introduced by Gray, 2019), one focus of the argument rested on the predominance of cognitive psychology and the displacement of smaller disciplines, such as anthropology and philosophy. Much less attention has been paid to the fact that another key player in the genesis of cognitive science has been withdrawing from the joint endeavor: artificial intelligence (AI) and computer science (Forbus, 2010; Goel, 2019). Although not framed in terms of the recent debate, the two topics in the current issue of *topiCS* are germane to this concern, as they both focus on cognitive modeling—arguably a signature approach of cognitive science and a natural link to AI.

The call for rapprochement is clearest in the first topic, *Cognition‐Inspired Artificial Intelligence*, edited by Daniel N. Cassenti (DEVCOM Army Research Laboratory), Vladislav D. Veksler (Caldwell University), and Frank E. Ritter (Pennsylvania State University). To showcase how cognitive science has not just benefitted from advances in AI, but can and should inspire AI development, Cassenti, Veksler, and Ritter bring together contributions from researchers actively using cognitive modeling to tackle a wide range of cognitive phenomena.

Incidentally, the other topic in this issue seconds this call for greater attention to and consideration of cognitive models by presenting spearheading work in this very field. For their topic, Terrence C. Stewart (National Research Council Canada) and Joost de Jong (Maastricht University) have assembled revised and expanded versions of the five best papers presented at last year's *19th International Conference on Cognitive Modeling*, a conference devoted to computational systems that are aimed at reflecting the internal processes of the mind. In their introduction, Stewart and de Jong point out how these papers, despite their diversity in content, still complement one another in terms of focus and approach: by refining and advancing computational models to better reflect empirical data, or by using such models to better explain data. Congratulations to their authors for their awards––we hope you continue the outstanding work you are doing!

In conclusion, we remind our readers that our publisher, Wiley, allows us to offer the Topic Editors’ introduction to their topic to all our readers as a free download.

*topiCS* encourages letters and commentaries on all topics, as well as proposals for new topics. Letters are not longer than two published pages (ca. 400–1000 words). Commentaries (between 1000 and 2000 words) are often solicited by Topic Editors prior to the publication of their topic, but they may also be considered after publication. Letters and commentaries typically come without abstracts and with few references, if any.

The Executive Editor and the Senior Editorial Board (SEB) are constantly searching for new and exciting topics for *topiCS*. Feel free to open communications with a short note to the Executive Editor (Andrea.Bender@uib.no) or a member of the SEB (for a list, see the publisher's homepage for *topiCS*: http://onlinelibrary.wiley.com/journal/10.1111/(ISSN)1756‐8765/homepage/EditorialBoard.html).
